# Therapeutic Effect of *Hedera helix* Alcoholic Extract Against Cutaneous Leishmaniasis Caused by *Leishmania major* in Balb/c Mice

**DOI:** 10.5812/jjm.9432

**Published:** 2014-04-01

**Authors:** Hossein Hooshyar, Safarali Talari, Fatemeh Feyzi

**Affiliations:** 1 Department of Parasitology, School of Medicine, Kashan University of Medical Sciences, Kashan, IR Iran; 2 Anatomical Science Research Center, Kashan University of Medical Sciences, Kashan, IR Iran

**Keywords:** Leishmania, Therapeutics, Plants, *Hedera*

## Abstract

**Background:**

Cutaneous leishmaniasis (CL) is common and endemic in many areas of Iran, caused by species of a protozoan parasite belonging to the genus *Leishmania*. There is not any effective vaccine against leishmaniasis; so, therapy is important for prevention and separation of disease. Herbal extract for treatment of CL is cost-effective, applicable topically to lesions, and can avoid the development of drug resistance.

**Objectives:**

The aim of this study was to evaluate the *in vivo* activity of an alcoholic extract of *Hedera helix* (a native Iranian plant) on the experimental ulcer of zoonotic CL in Balb/c mice.

**Materials and Methods:**

At least 5x l0^6^ promastigotes of *Leishmania major* (MHOM/64/IR/ER75) were inoculated subcutaneously in the tail base of Balb/c mice. Fifty six infected mice were distributed in four groups, two groups (16 mice for 20% alcoholic extract of *H. helix* and 13 for 70% extract) were used as experimental groups, one (15 mice) as placebo control (Control A), and one (12 mice) as negative control. Treatment effects of two concentrations were determined by comparison of placebo and nontreated groups via measuring the size of skin lesions and the number of parasitologically positive and negative mice after the therapy period.

**Results:**

This study showed that the main lesion size did not decrease significantly, or the small lesions did not completely disappear after treatment by *H. helix* alcoholic extract. Amastigotes counts (mean ± SD) of the skin lesions decreased in control A and 20% concentration groups, but in negative control and 70% concentration groups the number of parasites did not reduce.

**Conclusions:**

The present study did not support the *in vivo* antileishmanial effect of *H. helix* extract. We recommend further studies using major components of *H. helix,* especially hederasaponin (saponin K10), to investigate the antileishmanial effect of this plant on *L. major*.

## 1. Background

Leishmaniasis is endemic in 88 countries and about 350 million people are at risk. The prevalence of the disease is about 12 million worldwide (1). Clinical feature of the disease include cutaneous leishmaniasis (CL), mucocutaneous leishmaniasis (MCL), and visceral leishmaniasis (VL) (2, 3). CL is a group of diseases caused by several species of a protozoan parasite belonging to the genus *Leishmania*, transmitted by sand fly insect vector. The disease usually causes skin lesion on the exposed region of the body and leaves permanent scars. 

Annual incidence of CL is 1 to 1.5 million cases, of which 90% occurs in only seven countries: Afghanistan, Algeria, Brazil, Iran, Peru, Saudi Arabia and Syria (4). CL is a major health problem in many areas of Iran. This disease includes anthroponotic CL (ACL) caused by *Leishmania tropica* and zoonotic CL (ZCL) caused by *L. major* that are endemic in many different parts of Iran (3, 5). *L. major* is the etiological agent for ZCL, endemic in some regions, especially rural areas of 17 out of 31 provinces of Iran (5, 6). It is estimated that about 80% of the cases reported in this country are of ZCL form (6). Systemic treatment of ZCL is intralesional or systemic administration of antimony compounds (sodium stibogluconate and meglumine antimonies) (4). These compounds are parenteral and associated with significant side effects (7). Other disadvantages of these drugs are requirement for intramuscular or intralesion injection every day for a long period, toxicity, and the recent resistance development in regions such as India (8).

During the last decades, many attempts have been made to obtain effective new compounds especially herbal extracts, for treatment of CL that would be economical, applicable topically to the lesions, and could avoid resistance development (9). Plants contain a wide variety of metabolites such as tannins, terpenoids, alkaloids and flavonoids, found to have antimicrobial properties (10). Natural extracts of different plants such as Thyme, Yarrow, *Euphorbia* spp., *Gossypium herbacium*, *Berberis vulgaris*, *Alkanna tincturia* and *Peganum harmala* have been directly used on skin lesions as well as on the parasite in NNN medium (11-13). 

*Hedera helix* is a species of ivy, native to most of Eurasia, including Britain, south and east Mediterranean areas and Iran. It is an evergreen climbing plant, growing up to 20 – 30 m high where suitable surfaces (trees, cliffs, walls) are available, and also growing as ground cover where there are no vertical surfaces. Leaves of this plant are used in traditional medicine as bronchospasmolitic, secretolytic and anti-inflammatory, also as an anthelmintic, and as an agent to reduce fever and cause diaphoresis. Some studies showed the effectiveness of leaves extract of this plant in fighting the bacterial, protozoal and fungal infections (14-16). Eguale et al. recently showed that hydro-alcoholic extracts of *H. helix* had antihelmenthic activity against *Haemonchus contortus* *in vitro* and *in vivo* (17). *H. helix* is an endemic plant species growing in many regions. In Iran, this plant grows mainly in Kordestan and Shahrood cities as well as central, western, and eastern areas (18).

## 2. Objectives

The present study was an experimental research performed to determine and evaluated the effects of different concentrations of *H. helix* extract on Balb/c murine model infected by active promastigote of *L. major*.

## 3. Materials and Methods

This experimental and *in vivo* study was carried out on 90 Balb/c mice weighing approximately 30 g and 4 - 6 weeks of age (purchased from Pasteur Institute, Karaj, Iran). This study was performed according to the ethical standards and approved by ethics committee of deputy of research of Kashan University of Medical Sciences (no. 8304). The standard virulence strain of *L. major* (MHOM/64/IR/ER75) was obtained from the School of Public Health, Tehran University of Medical Sciences, cultured in RPMI-1640 (Gibco, Australia) supplemented with 15% inactivated fetal calf serum (FCS, Gibco, Australia). At least 5 × l0^6^ promastigotes, harvested at the stationary phase of the culture, were inoculated subcutaneously in the tail base of the mice. After 40 days, nodules and ulcers appeared on 56 inoculated mice. Fifty six infected mice were distributed in four groups, two (16 mice for 20% extract and 13 for 70% extract) were used as experimental groups, one (15 mice) as placebo control (control A) and one (12 mice) as negative control. Leaves of *H. helix* were acquired from Kashan City, central Iran. Afterwards, the leaves were washed, dried, and extracted by Soxhlet apparatus and solved in ethanol 80% (percolation method) (19) in Barij Essence Pharmaceutical Company.

Before using the plant extract for treatment, diameters of each lesion were measured using scale and vernier calipers. Impression smears were prepared from the lesions; the slide were fixed with absolute methanol, stained by Giemsa stain, and examined by light microscopy (1000x). Two concentrations (20% and 70%) of the *H. helix* extract were tested on the CL lesions of two groups of mice by rubbing on the local lesion two times a days, for 30 days. Control group A received ethanol 80% and negative control groups received no treatment. Treatment effects of two concentrations were determined through comparing the placebo and non-treated groups by measuring the size of the skin lesions and the number of parasitologically positive and negative mice after the therapy period. The results were analyzed as mean ± SE (standard error of mean). Paired t-test, ANOVA and Fisher tests were used to compare the treated and untreated groups. Data were considered statistically significant at P < 0.05.

## 4. Results

**Figure 1.**
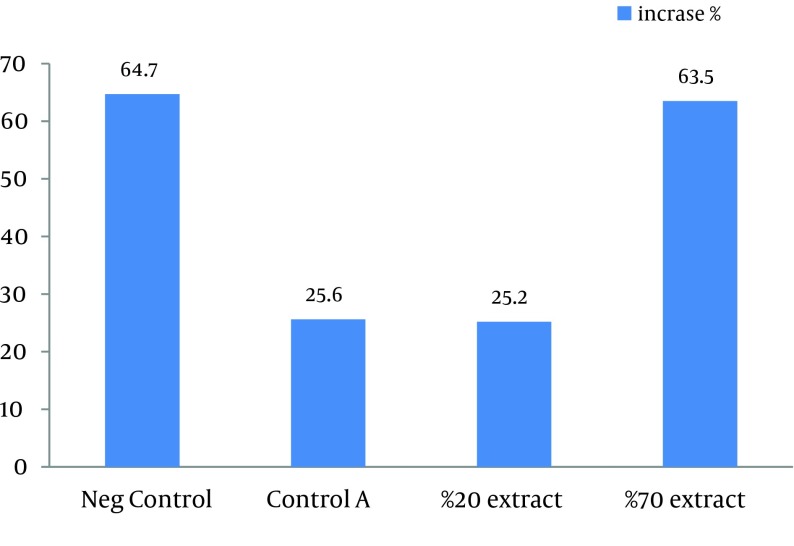
Diameter Increase Percentage of CL Ulcers of Mice Induced by *L. major*, Treated by Different Concentrations of *H. helix*

This study showed that in both 20% and 70% concentrations of alcoholic extracts of *H. helix,* the mean lesion size increased at the end of the treatment. Sizes of lesions in both groups did not significantly increase in comparison with the control A groups (treated with ethanol 80%). Statistically significant increase in the size of ulcers was observed in negative control group compared with other three groups (P < 0.03). Results of this study showed that the main lesion size did not decrease significantly or small lesions did not completely disappear after treatment by *H. helix* alcoholic extracts (Table 1 and Figure 1). Amastigotes counts (mean ± SD) of the skin lesions decreased in control A and 20% concentration groups, but the number of parasites did not reduce in negative control and 70% concentration groups. Direct smears of mice in the microscopic studies were positive after 1 month of treatment in all control and intervention groups.

**Table 1.** Therapeutic Effects of Different Concentrations of *H. helix* on the Average Diameters of CL Ulcers Induced by *L. major* in Balb/c Mice ^a^

Ulcer Diameter	Before the Treatment, mm	After the Treatment, mm	Increase, %
**Negative control**	5.15 ± 2.21	8.48 ± 3.59	64.7
**Control A**	6.69 ± 2.89	8.99 ± 2.84	25.6
**20% Extract**	7.98 ± 3.o1	9.99 ± 3.85	25.2
**70% Extract**	6.96 ± 2.22	11.38 ± 3.68	63.5

^a^ All data are presented in Mean ± SD.

## 5. Discussion

The purpose of this study was to examine the effect of alcoholic *H. helix* extract on Balb/c murine model mice, infected by active promastigote of *L. major*. According to the results of this study, two concentrations of *H. helix* extract (20% and70%) did not have any significant positive correlation with decrease of *L. major* ulcer diameter in Balb/c murine model. That was possibly due to several factors such as effectless of major active components of *H. helix* on this parasite. The second possible explanation for no significant effect of this plant extract on mice ulcer could be that *Leishmania* is an intracellular parasite and some factors such as genetic of the reservoir and the efficacy of cell-mediated immunity can hamper the influences of *H. helix* major components on the infected cells. This was in agreement with the previous research stating that leaves of *H. helix* contain the “emetine” compound, which is an amoebicidal alkaloid, and also triterpene saponins, which are effective against liver flukes, molluscs, intestinal parasites and fungal infections, all of which are extracellular parasites (14, 15).

Our findings were in disagreement with those of Talari et al. in which, 100 and 50 mg/mL of *H. helix* extract could kill all promastigotes of *L. major* *in vitro* (18). This difference of findings may be due to different methods and concentration of the plant extract in the two studies. The previous research used *in vitro* approach, eliminating the effect of immunoresponse of live hosts. In other words, promastigotes of *Leishmania* are extra cellular parasite, while amastigotes are obligate intracellular parasites. 

Majester-Savornin showed that an extract of *H. helix* contains 60% saponic complex (CS 60), bidesmosides hederasaponin B, C and D (saponin K10), their corresponding monodesmosides α-,β-, δ-hederin, and hederagenin. CS 60 and bidesmosides have shown no effects. Monodesmosides were found to be as effective on promastigotes of *L. infantom* and *L. tropica*. Against amastigote forms, only hederagenin exhibited a significant activity (20). However, further studies are required to investigate the activity of hederagenin on *L. major* amastigotes. Some studies have shown that some of Iranian and foreign medical plants have antileishmanial effects. For example, a study conducted by Manjili et al. showed that some Iranian medical plant including *Caesalpinia gilliesii*, *Satureia hortensis*, *Carum copticum* heirm, and *Thymus migricus*, displayed high antileishmanial activity and were toxic against this parasite both *in vitro* and *in vivo* (21). 

A recent study by Gharavi et al. indicated that garlic extract could destroy the amastigotes of *L. major* due overexpression of iNOS and IFNƳ genes in macrophages (22). Another study showed that the mixture of of thyme and yarrow alchoholic extracts had proper effects on treatment of cutaneous lesions of *L. major* in Balb/c infected mice (13). However, my previous study showed the *in vitro* antileishmanial effect of *H. helix* extract, as a definite yield, but the present study did not support this result and had contradiction with others studies. We recommend further studies using the major components of *H. helix* especially hederagenin, hederasaponin B, C and D (saponin K10), to investigate the antileishmanial effects of this plant on *L. major*.
